# Patterns, Severity, and Outcomes of Solid Organ Injuries in Abdominal Trauma: A Prospective Observational Study

**DOI:** 10.7759/cureus.106880

**Published:** 2026-04-12

**Authors:** Sumit Sharma, Vivek Katiyar, Avinash Kumar, Muskan Dugar, Shashi P Mishra, Satyanam Kumar U Bhartiya

**Affiliations:** 1 General Surgery, Institute of Medical Sciences, Banaras Hindu University, Varanasi, IND

**Keywords:** aast grading, blunt abdominal trauma, non-operative management, shock index, solid organ injury

## Abstract

Background: Solid organ injuries in blunt abdominal trauma cause significant morbidity and mortality, necessitating early severity assessment.

Objectives: The primary aim is to evaluate the pattern and severity of solid organ injuries after abdominal trauma. The secondary objectives of the study are to correlate injury severity with clinical, biochemical, and radiological parameters and to evaluate management strategies and short-term outcomes.

Method: This prospective observational study included patients presenting with blunt abdominal trauma and radiologically confirmed solid organ injuries. Injuries to the liver, spleen, kidney, and pancreas were graded according to the American Association for the Surgery of Trauma (AAST) classification. Clinical parameters, shock index, laboratory values, imaging findings, and management strategies were recorded and analyzed.

Results: Young males predominated, and road traffic accidents were the leading cause of injury. The liver and spleen were the most frequently injured organs. Higher AAST grades were associated with increased shock index values (>0.9), greater hemodynamic instability, and significant derangement of biochemical parameters, including elevated serum transaminases in liver injury, declining hemoglobin levels in splenic injury, raised creatinine in renal injury, and elevated amylase and lipase in pancreatic injury. Non-operative management was successful in most hemodynamically stable patients, whereas operative intervention was required primarily in patients with persistent instability or failure of conservative management.

Conclusion: Blunt abdominal solid organ injuries are commonly managed successfully with non-operative strategies. The shock index is a useful predictor of injury severity, and combined clinical, biochemical, and radiological assessment aids in timely decision-making.

## Introduction

Abdominal trauma can affect every organ system and major vascular structure with potentially devastating effects. When we consider abdominal solid organ injury, we usually think of the liver, spleen, and kidneys. However, all abdominal organs, including the pancreas and adrenal glands, may be involved. Hepatic trauma is more commonly associated with venous bleeding rather than arterial injury. Stable venous injury is often managed conservatively; however, when the patient is hemodynamically unstable due to venous hepatic injury, operative management should be the first-line therapy. When the injury is arterial, endovascular therapy should be initiated. In the setting of blunt abdominal trauma (BAT), renal and pancreatic injuries are less frequent than hepatic and splenic injuries [1]. Early and rapid diagnosis is necessary to reduce morbidity.

The management of patients with solid organ injuries has changed since the introduction of technically advanced imaging tools such as ultrasonography and multiple scan computerized tomography; interventional radiological techniques; and modern intensive care units [2]. The advent of newer imaging techniques with high-resolution CT scanners has enabled clinicians to accurately diagnose the extent of intra-abdominal organ injury [3].

In the setting of blunt trauma to the abdomen, the liver and spleen are the most commonly injured solid organs; kidney injuries occur less frequently. Such injuries have been successfully managed non-operatively, and there is a growing interest in angioembolization (AE) as an important tool in the trauma surgeon’s armamentarium. As such, AE has become an integral component of multidisciplinary algorithms for achieving hemostasis in trauma patients [4].

Laparoscopy has been proven useful in the treatment of complications after non-operative management (NOM) for severe hepatic trauma, when interventional radiology (e.g., percutaneous drainage) fails. The Eastern Association for the Surgery of Trauma (EAST) guidelines on NOM for blunt hepatic trauma state that adjunctive therapies such as angiography, percutaneous drainage, endoscopy/endoscopic retrograde cholangiopancreatography (ERCP), and laparoscopy remain important adjuncts to NOM of hepatic injuries [5].

Non-operative treatment avoids surgical risks such as anesthesia-related complications, iatrogenic injury, infections, hernias, adhesions, prolonged hospital stays, higher costs, and increased morbidity and mortality. It is safe in hemodynamically stable patients without peritoneal signs, and even selected high-grade solid organ injuries can be managed with close medical follow-up [6].

The primary objective of this study is to evaluate the pattern and severity of solid organ injuries following abdominal trauma. The secondary objectives are to correlate injury severity with clinical, biochemical, and radiological parameters and to evaluate management strategies and short-term outcomes.

## Materials and methods

The study was conducted in the Department of Surgery in collaboration with the Department of Radiodiagnosis and Department of Pathology at a tertiary care center. The duration of the study was for two years, from September 2019 to August 2021. Ethical clearance was obtained from the Institutional Ethics Committee before starting the study. The study was conducted and reported in accordance with the STROBE (Strengthening the Reporting of Observational Studies in Epidemiology) guidelines. A total of 150 patients were selected for the study based on the inclusion and exclusion criteria. A consecutive sampling method was employed in this study. All patients fulfilling the inclusion criteria and presenting during the study period were consecutively enrolled until the desired sample size was achieved. 

Inclusion criteria

All patients aged 15-60 years presenting with abdominal trauma, including both blunt and penetrating injuries, were included in the study.

Exclusion criteria

Patients with polytrauma, significant co-morbidities or pregnancy, known liver, spleen, kidney or pancreatic disease, bleeding disorders, and those with missed hollow viscus injuries were excluded from the study.

All patients were evaluated using primary and secondary surveys as per the ATLS protocol, along with a detailed history and clinical examination to determine eligibility for inclusion. The shock index was calculated at presentation using the formula SI = HR/SBP, where HR is the heart rate and SBP is the systolic blood pressure. Patients underwent serial, vigilant clinical and biochemical monitoring throughout their hospital stay. The primary outcome was defined as injury severity and management strategy, while secondary outcomes such as need for operative intervention, complications, and mortality were also considered.

Biochemical evaluation included complete blood count, liver function tests, renal function tests, prothrombin time and prothrombin time-international normalized ratio (PT-INR), urine routine microscopy and culture sensitivity, serum amylase, serum lipase, and serum electrolytes. These tests were done at admission, at six hours, and at 24 hours post presentation. Radiological evaluation comprised FAST, contrast-enhanced CT (CECT) abdomen, and ultrasonography. All hemodynamically stable patients underwent CECT using a 64-slice multidetector CT scanner following a standardized trauma protocol and were interpreted by a dedicated team of radiologist at the trauma center. Solid organ injuries were graded according to the American Association for the Surgery of Trauma (AAST) injury grading system. Clinical, radiological, and biochemical findings were correlated and analyzed (Video 1).

**Video 1:** CECT of the abdomen with angiographic protocol (3 mm thin-slice, venous phase) demonstrating Grade III hepatic injury and Grade IV splenic injury, along with perihepatic and perisplenic fluid collections. CECT: contrast-enhanced CT.

Based on the assessments, patients were categorized as hemodynamically unstable, hemodynamically stable, or hemodynamically unstable but responsive to primary resuscitation. The operational definitions used in the present study are summarized in Table 1. All hemodynamically unstable patients underwent exploratory laparotomy. Hemodynamically stable patients and responders were managed non-operatively with close monitoring of vital and biochemical parameters in the Trauma ICU.

**Table 1:** Hemodynamic definitions used in the study. SBP: systolic blood pressure.

Parameter	Definition
Hemodynamically stable	SBP ≥ 90 mmHg, heart rate < 100 bpm, and shock index ≤ 0.9 with adequate response to initial fluid resuscitation
Hemodynamically unstable	SBP < 90 mmHg, heart rate > 100 bpm, or shock index > 0.9 despite initial fluid resuscitation
Transient responder	Initial improvement in blood pressure after fluid resuscitation followed by recurrent hypotension or tachycardia
Non-responder	Persistent hypotension (SBP < 90 mmHg) and tachycardia despite adequate fluid resuscitation requiring urgent operative or interventional management

NOM was considered to have failed in cases of persistent hemodynamic instability, significant drop in hemoglobin (≥2 g/dL within 24 hours requiring blood transfusion), development of peritonitis, or radiological evidence of ongoing hemorrhage requiring operative intervention. Such patients underwent repeat ultrasonography followed by diagnostic laparoscopy and drain placement. ICU monitoring was continued after intervention or in unstable patients. All patients were followed up in outpatient clinics for 12 weeks following the initial trauma. Complications such as biloma and liver abscess were treated with ultrasound-guided percutaneous drainage; bile leak and pancreatic duct injuries were managed with ERCP and stent placement; post-traumatic hepatic artery pseudoaneurysm was treated with digital subtraction angiography (DSA)-guided AE; and pancreatic pseudocysts were managed non-operatively.

Diagnostic laparoscopy was performed in patients with failure of NOM, as indicated by clinical signs such as tachycardia, hypotension, peritonitis, significant abdominal distension, and biochemical evidence of a falling hematocrit. Exploratory laparotomy was performed in all hemodynamically unstable patients after primary resuscitation. Postoperatively, all patients were shifted to the Trauma ICU for continued monitoring until hemodynamic stability was achieved. All patients were followed up in the OPD for up to 12 weeks post-trauma.

Data were collected and analyzed using IBM SPSS Statistics for Windows, Version 24.0 (Released 2016; IBM Corp., Armonk, New York, United States). Continuous variables were expressed as mean ± standard deviation, while categorical variables were presented as frequencies and percentages. The Student’s t-test was used to compare continuous variables between groups, and the chi-square test or Fisher’s exact test was used for categorical variables. A p-value <0.05 was considered statistically significant. Receiver operating characteristic (ROC) curve analysis was performed to assess the diagnostic accuracy of the shock index (>0.9) for predicting severe solid organ injury (AAST grades IV-V).

## Results

In the above study, the majority of patients belonged to the BAT group, with 132 of 150 cases (88%), while 18 of 150 cases (12%) belonged to the penetrating abdominal trauma group. The ratio of blunt to penetrating abdominal trauma was 7.3:1 (Table 2). Table 3 summarizes the relationship between the shock index and the severity of solid organ injury graded according to the AAST classification.

**Table 2:** Type of injury. Distribution of patients according to mechanism of abdominal trauma (blunt vs penetrating). Values are presented as number (%). BAT: blunt abdominal trauma.

Type of injury	Number (%)
BAT	132 (88.0)
Penetrating abdominal trauma	18 (12.0)

**Table 3:** Relationship between solid organ injury grade and shock index. Association between AAST injury grade and shock index (SI = heart rate/systolic blood pressure) for liver, spleen, kidney, and pancreas. Values are presented as number of patients. “–” indicates grade not applicable or not observed. AAST: American Association for the Surgery of Trauma.

Organ	Shock index	Grade I	Grade II	Grade III	Grade IV	Grade V	Total
Liver	<0.9	8	14	20	2	0	44
	>0.9	2	8	11	15	1	37
	Total	10	22	31	17	1	81
Spleen	<0.9	5	15	12	2	0	34
	>0.9	0	2	12	24	1	39
	Total	5	17	24	26	1	73
Kidney	<0.9	–	6	8	1	–	15
	>0.9	–	1	7	3	–	11
	Total	–	7	15	4	–	26
Pancreas	<0.9	–	2	2	0	–	4
	>0.9	–	1	2	5	–	8
	Total	–	3	4	5	–	12

**Figure 1:**
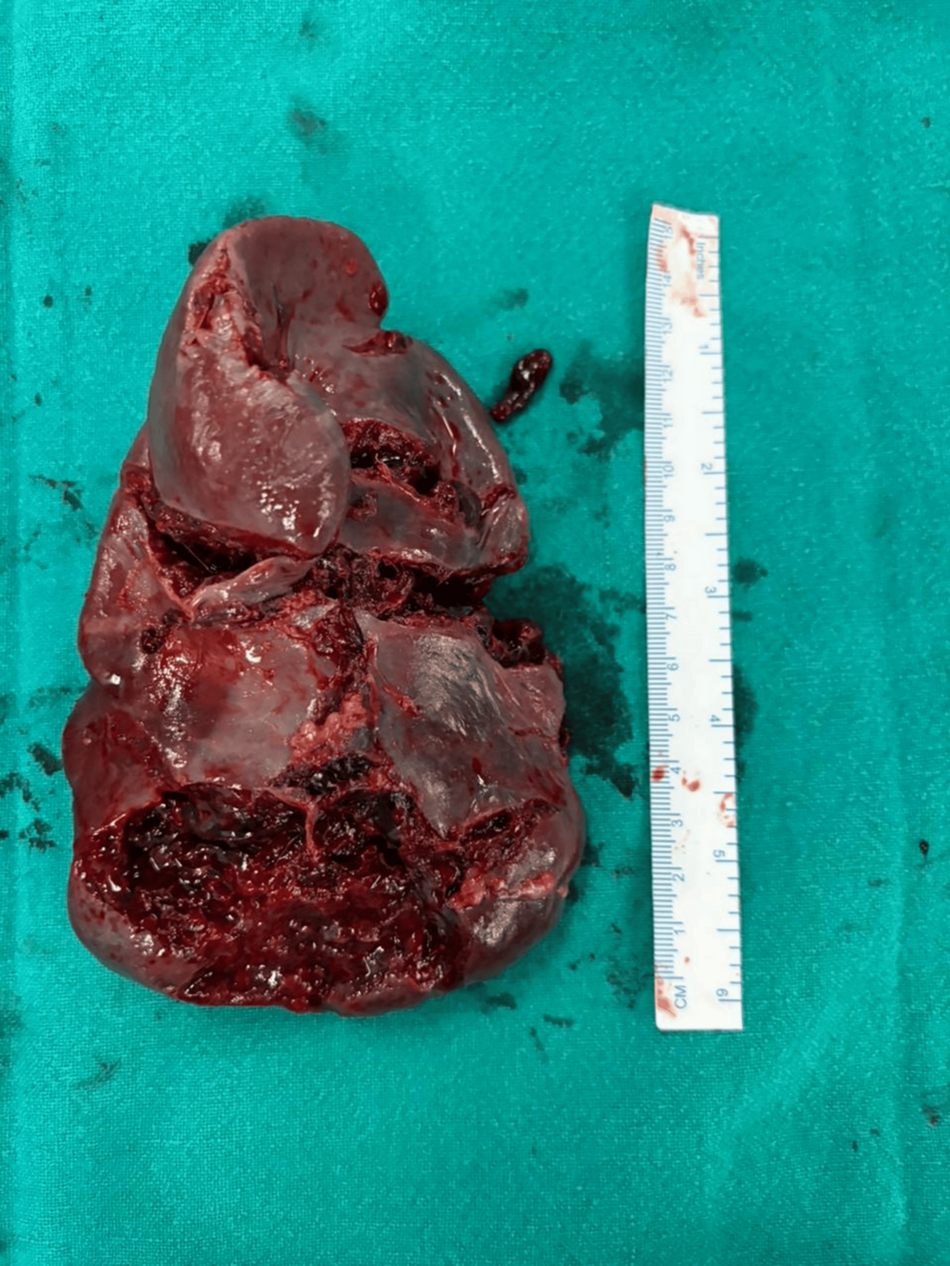
Splenectomy specimen in AAST grade V spleen injury, involving hilum. AAST: American Association for the Surgery of Trauma.

**Figure 2:**
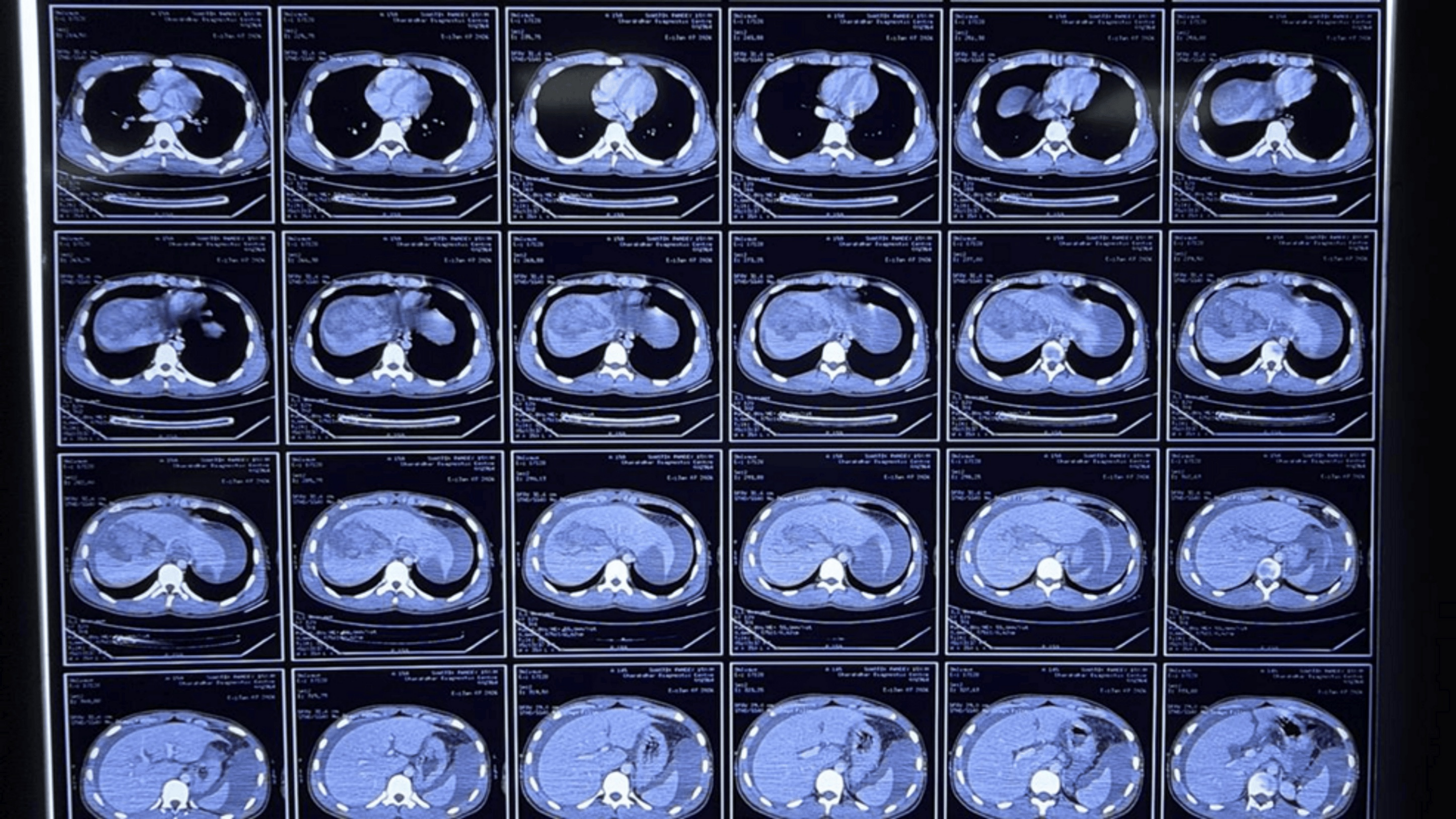
CECT whole abdomen depicting grade III liver injury with perihepatic and perisplenic collections following blunt trauma to abdomen. CECT: contrast-enhanced CT.

**Figure 3:**
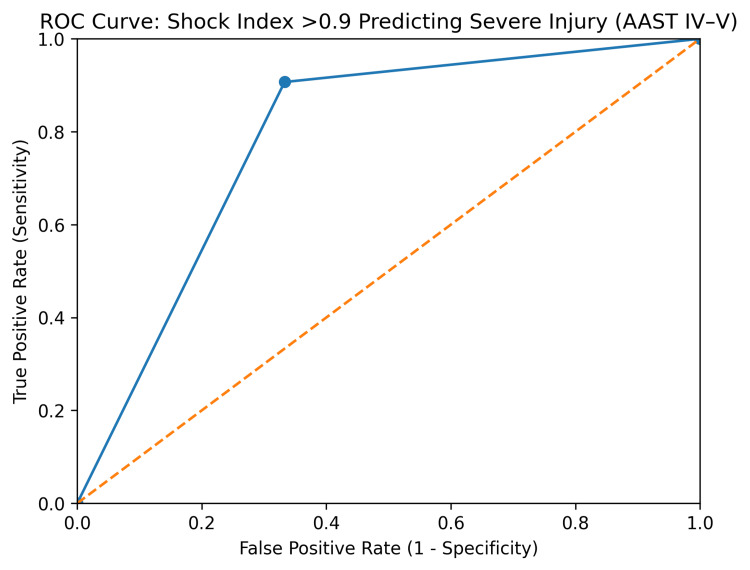
ROC curve demonstrating the diagnostic performance of the shock index >0.9 in predicting severe solid organ injury (AAST grades IV–V). The estimated AUC was 0.79, indicating acceptable discriminative ability. ROC: receiver operating characteristic; AUC: area under the ROC curve.

Among patients with liver injury, a greater proportion had a shock index <0.9 (54.3%), predominantly in lower injury grades (I-III), whereas higher grades (IV-V) were more frequently associated with a shock index >0.9. In splenic injuries, more than half of the patients (53.4%) demonstrated a shock index >0.9, with a clear trend toward increasing shock index values in higher grades, particularly grades IV and V (Figure 1). Renal injuries showed a relatively balanced distribution, although a higher shock index (>0.9) was observed more commonly in patients with grade III and IV injuries. In pancreatic injuries, the majority of patients (66.7%) had a shock index >0.9, with higher grades showing a stronger association with elevated shock index. Overall, increasing injury grade across solid organs was associated with a higher shock index, suggesting a correlation between hemodynamic instability and injury severity (Figure 2). Receiver operating characteristic (ROC) analysis was performed to evaluate the diagnostic performance of the shock index (>0.9) in predicting severe solid organ injury (AAST grades IV-V). Shock index >0.9 demonstrated a sensitivity of 90.7% and specificity of 66.7% for identifying severe injury. The positive predictive value was 51.6% and the negative predictive value was 94.8%. The estimated area under the ROC curve (AUC) was 0.79, indicating acceptable discriminative ability of the shock index for predicting high-grade injury (Figure 3).

The biochemical parameters in solid organ injuries according to AAST grade are shown in Table 4. The table demonstrates a progressive derangement of biochemical parameters with increasing injury grade across solid organs. Liver injuries showed rising transaminases, alkaline phosphatase, PT, and INR with higher grades. Splenic injuries were associated with a progressive decline in hemoglobin levels over time, most pronounced in higher grades. Renal injuries demonstrated increasing creatinine and urea levels with advancing grade, while pancreatic injuries showed a marked elevation of serum amylase and lipase in higher-grade injuries. Management of liver injury is shown in Table 5. The table shows that 2 of 17 patients (11.8%) with AAST grade IV injuries are hemodynamically unstable at presentation and 1 patient with AAST grade V liver injury remained hemodynamically unstable even after initial resuscitation and underwent operative management.

**Table 4:** Biochemical parameters in solid organ injuries according to AAST grade. Comparison of organ-specific biochemical parameters across increasing AAST grades. Values are presented as mean ± standard deviation. AAST: American Association for the Surgery of Trauma; ALT: alanine aminotransferase; AST: aspartate aminotransferase; ALP: alkaline phosphatase; PT: prothrombin time; INR: international normalized ratio.

Organ	Parameter	Grade I (mean ± SD)	Grade II (mean ± SD)	Grade III (mean ± SD)	Grade IV (mean ± SD)	Grade V (mean ± SD)
Liver	ALT (IU/L)	329 ± 92	305 ± 220	1018 ± 818	1482 ± 849	3210 ± 0
	AST (IU/L)	220 ± 58	200 ± 184	710 ± 647	1594 ± 1005	3210 ± 0
	ALP (IU/L)	125 ± 58	250 ± 65	423 ± 222	299 ± 168	1789 ± 0
	PT (seconds)	14.67 ± 1.03	15.54 ± 0.87	17.02 ± 1.52	17.38 ± 1.61	19.00 ± 0
	INR	1.00 ± 0.00	1.19 ± 0.12	1.41 ± 0.40	1.50 ± 0.36	2.00 ± 0
Spleen	Hb at presentation (g/dL)	–	10.52 ± 2.01	10.15 ± 1.65	9.23 ± 2.04	–
	Hb at 6 hours (g/dL)	–	9.46 ± 1.58	9.11 ± 0.93	8.06 ± 1.20	–
	Hb at 24 hours (g/dL)	–	10.43 ± 1.10	9.21 ± 1.38	9.12 ± 2.06	–
Kidney	Creatinine (mg/dL)	–	1.1 ± 0.0	2.1 ± 0.9	2.9 ± 0.8	–
	Urea (mg/dL)	–	70 ± 0	81 ± 45	92 ± 11	–
Pancreas	Amylase (IU/L)	–	783 ± 147	1586 ± 513	1563 ± 0	–
	Lipase (IU/L)	–	595 ± 49	1321 ± 158	1739 ± 0	–

**Table 5:** Management of liver injury. Distribution of operative and NOM strategies for liver injuries stratified by AAST grade. Values are presented as number (%). NOM includes hemodynamically stable patients and responders to resuscitation. AAST: American Association for the Surgery of Trauma; NOM: non-operative management.

Liver injury (n=81) AAST-2018	n	NOM (hemodynamically stable+responders)	Operative management (hemodynamically unstable)
Grade I	10	10+0 (12.3%)	0
Grade II	22	16+6 (27.3%)	0
Grade III	31	21+10 (32.3%)	0
Grade IV	17	1+14 (88.2%)	2 (11.8%)
Grade V	1	0+0	1 (100%)

The majority of patients with AAST grades I, II, III and IV were managed non-operatively. Failures of NOM in liver injury are shown in Table 6. The table summarizes the outcomes of NOM for liver injuries across 81 cases, specifically identifying the frequency of management failure categorized by the AAST-2018 grading system. The management of non-operative failure cases is shown in Table 7. The table shows that 6 of 8 (75%) patients with AAST grade III and IV liver injuries were managed by diagnostic laparoscopy and drain placement, and 2 of 8 (25%) patients with AAST grade IV liver injury underwent AEs. The management of splenic injury is shown in Table 8.

**Table 6:** Failures of NOM in liver injury. Frequency of failure among patients initially managed non-operatively for liver trauma across different AAST grades. Values are presented as number (%). AAST: American Association for the Surgery of Trauma; NOM: non-operative management.

Liver injury (n=81) AAST-2018	NOM (hemodynamically stable+responders)	Failures
Grade I	10+0 (12.3%)	0
Grade II	16+6 (27.3%)	0
Grade III	21+10 (32.3%)	0+3
Grade IV	1+14 (88.2%)	0+5
Grade V	0+0	0

**Table 7:** Management of non-operative failure cases (n=8) of liver injury. Interventions performed in patients with failed NOM of liver injury, including diagnostic laparoscopy with drainage and AE. Values are presented as number of patients. AAST: American Association for the Surgery of Trauma; AE: angioembolization; NOM: non-operative management.

Liver injury grade (AAST-2018)		Management
Grade III	Grade IV	
3	3	Diagnostic laparoscopy with drain placement
0	2	Angioembolization

**Table 8:** Management of splenic injury. Distribution of operative and non-operative treatment modalities for splenic injuries based on injury severity. Values are presented as number (%). AAST: American Association for the Surgery of Trauma; NOM: non-operative management.

Splenic injury (n=73) AAST-2018	n	NOM (hemodynamically stable+responders)	Operative management (hemodynamically unstable)
Grade I	5	5+0 (100%)	0
Grade II	17	17+0 (100%	0
Grade III	24	6+16 (91.7)	1+1 (8.3%)
Grade IV	26	1+18 (73.1%)	3+4 (26.9%)
Grade V	1	0+0	1 (100%)

Management of renal injury is shown in Table 9. The table shows that all with renal injury (AAST grade II, III, IV) are hemodynamically stable or became stable after initial resuscitation and managed non-operatively. Management of pancreatic injury is shown in Table 10. The table shows that 1 of 4 (25%) patients with AAST grade III pancreatic injury and 4 of 5 (80%) patients with AAST grade IV pancreatic injury are hemodynamically unstable at presentation even after initial resuscitation and managed operatively.

**Table 9:** Management of renal injury. Treatment outcomes of renal trauma categorized by AAST grade. Values are presented as number (%). All patients were managed non-operatively. AAST: American Association for the Surgery of Trauma; NOM: non-operative management.

Renal injury (n=26) AAST-2018	n	NOM (hemodynamically stable+responders)	Operative management (hemodynamically unstable)
Grade I	0	0+0	0
Grade II	7	7+0 (100%)	0
Grade III	15	14+1 (100%)	0
Grade IV	4	2+2 (100%)	0
Grade V		0+0	0

**Table 10:** Management of pancreatic injury. Operative and NOM patterns for pancreatic injuries stratified by AAST grade. Values are presented as number (percentage). AAST: American Association for the Surgery of Trauma; NOM: non-operative management.

Pancreatic injury (n=12) AAST-2018	n	NOM (hemodynamically stable + responders)	Operative management (hemodynamically unstable)
Grade I	0	0+0	0
Grade II	3	2+1 (100%)	0
Grade III	4	1+2 (75%)	1 (25%)
Grade IV	5	0+1 (20%)	4 (80%)
Grade V	0	0+0	0

All patients of AAST grade II pancreatic injury and 3 of 4 (75%) patients with AAST grade III pancreatic injury are hemodynamically stable or responded to initial resuscitation and are managed non-operatively. Mortality in solid organ injury is shown in Table 11. The table shows that no mortality noted in patients who were managed non-operatively and 3 of 150 (2%) patients have in-hospital mortality noted, and all these were managed operatively. Complication of liver injury is shown in Table 12. Patients with liver injury developed bile leak in post-op, which was managed by ERCP and stenting. In patients who were managed non-operatively, five patients with liver injury developed biloma, which was managed by USG-guided percutaneous drainage. Four patients with liver injury developed liver abscess in follow-up, which was managed by USG-guided percutaneous drainage. Two patients developed hepatic aneurysm, which was managed by AE. Complication in pancreatic injury is shown in Table 13. The table shows that one patient with pancreatic injury who was managed non-operatively developed pancreatic pseudocyst, which was managed conservatively, and two patients with pancreatic injury developed pancreatic fistula who were managed operatively, with pancreatic fistula management done by ERCP with stenting.

**Table 11:** Mortality in solid organ injury in abdominal trauma. Comparison of in-hospital mortality between non-operative and operative management groups. Values are presented as number (percentage). NOM: non-operative management.

Management	n =150	In-hospital mortality
NOM	132 (88%)	0
Operative management	18 (12%)	3

**Table 12:** Complications of liver injury. Post-treatment complications observed in patients with liver injury and corresponding management strategies. Values are presented as number of patients. AE: angioembolization; ERCP: endoscopic retrograde cholangiopancreatography.

Complication	Non-operatively managed patients	Operatively managed patients	Further management in non-operatively managed patients	Further management in operatively managed patients
Bile leak		2		ERCP and stenting
Biloma	5	0	Ultrasonic graphic Percutaneous drainage	-
Liver abscess	4	0	Ultrasonic graphic Percutaneous drainage	-
Hepatic artery aneurysm	2	0	AE	-

**Table 13:** Complications of pancreatic injury and their management. Complications following pancreatic injury and the respective therapeutic interventions. Values are presented as number of patients.

	Non-operatively managed patients	Operatively managed patients	Further management in non-operatively managed patients	Further management in operatively managed patients
Pancreatic pseudo cyst	1	0	Conservatively	
Pancreatic fistula	0	2		ERCP with stenting

## Discussion

Trauma, derived from the Greek word meaning "wound," refers to physical injury caused by the application of external force, with severity depending on the magnitude of impact. BAT accounts for nearly 75% of all BAT cases and is far more common than penetrating abdominal trauma, most frequently resulting from motor vehicle collisions [7].

BAT accounted for the majority of cases (88%) in this study and predominantly affected young males, with 40% of patients in the 15- to 25-year age group and a male-to-female ratio of 5.8:1. These findings are consistent with previous studies by Bolandparvaz et al. [8] and Mukhopadhyay [9]. Road traffic accidents were the commonest mechanism of injury, as also reported by Isenhour and Marx [7]

The liver was the most frequently injured solid organ (54%), followed by the spleen (48.7%), kidney (17.3%), and pancreas (8%), which is comparable to the distribution reported by Arumugam et al. [10], Baygeldi et al. [11], and El-Menyar et al. [12]. Combined solid organ injuries most commonly involved the liver and spleen (12%).

AAST grade III injuries were the most common for liver (38.3%) and spleen (32.9%). Higher-grade injuries were associated with greater hemodynamic instability. The shock index (SI) >0.9 was observed in 45.7% of liver injuries, 53.4% of splenic injuries, 42.3% of renal injuries, and 66.7% of pancreatic injuries. Notably, SI >0.9 was present in 82.3% of AAST grade IV liver injuries, 92.3% of grade IV splenic injuries, 75% of grade IV renal injuries, and all patients with grade IV pancreatic injury, supporting SI as a reliable marker of injury severity [12].

Biochemical parameters showed a clear correlation with injury grade. The mean ALT increased from 328.8 ± 92.3 IU/L in AAST grade I liver injury to 1482.3 ± 849.1 IU/L in grade IV and 3210 IU/L in grade V. The mean AST similarly rose from 220.3 ± 57.9 IU/L in grade I to 1594.2 ± 1005.4 IU/L in grade IV, with a statistically significant association (p = 0.001). Prothrombin time increased from 14.67 ± 1.03 seconds in grade I to 17.38 ± 1.61 seconds in grade IV, while INR rose from 1.0 to 1.5, indicating worsening hepatic dysfunction with increasing injury grade.

In splenic injuries, mean hemoglobin at presentation declined from 10.52 ± 2.01 g/dL in grade II to 9.23 ± 2.04 g/dL in grade IV, with a further fall at six hours (8.06 ± 1.20 g/dL in grade IV; p = 0.03). Renal injuries showed rising creatinine levels from 1.1 ± 0.0 mg/dL in grade II to 2.9 ± 0.8 mg/dL in grade IV. Pancreatic injuries demonstrated increasing enzyme levels, with mean amylase rising from 783 ± 147 IU/L in grade II to 1563 IU/L in grade IV, and lipase increasing from 595 ± 49 IU/L to 1739 IU/L, consistent with reports by Hosseininejad and Bozorgi [13].

NOM was successful in most hemodynamically stable patients, including those with higher-grade injuries. Failure of NOM was observed in 9.7% of AAST grade III and 33.3% of grade IV liver injuries, primarily in patients with persistent hemodynamic instability, consistent with outcomes reported by Hagiwara et al. [14], Mingoli et al. [15], and Singh et al. [16].

This study has several limitations. First, it was conducted at a single tertiary care center, which may introduce referral bias and limit generalizability to other settings. Second, no formal sample size calculation was performed, and the sample size was determined by the number of eligible patients presenting during the study period. Third, the penetrating trauma subgroup was relatively small and was not analyzed separately, which may influence interpretation of overall outcomes. Fourth, the analysis was primarily descriptive and did not include multivariable regression to adjust for potential confounding factors such as age, mechanism of injury, or associated injuries. Finally, interobserver variability in AAST injury grading was not formally assessed.

## Conclusions

BAT predominantly affects young males and remains the most common mechanism of solid organ injury. In this study, the liver and spleen were the most frequently injured organs, with higher AAST grades associated with increasing hemodynamic instability, elevated shock index, and progressive derangement of biochemical parameters.

The shock index demonstrated a significant association with the severity of abdominal solid organ injuries and may serve as a useful bedside indicator during initial trauma assessment. However, larger multicentric studies with comprehensive statistical modeling are required to validate its predictive utility. Biochemical markers such as serum transaminases, hemoglobin trends, creatinine, amylase, and lipase showed a clear relationship with organ-specific injury severity and can aid in early diagnosis, risk stratification, and monitoring.

NOM was successful in the majority of hemodynamically stable patients, including selected high-grade injuries, reinforcing its role as the standard of care in appropriately selected cases. Operative intervention was primarily required in patients with persistent hemodynamic instability or failure of conservative management. Overall, a combined assessment of clinical status, shock index, imaging findings, and biochemical parameters is essential for optimal decision-making and improves outcomes in patients with abdominal solid organ injuries.
